# Metatranscriptomic exploration of microbial functioning in clouds

**DOI:** 10.1038/s41598-019-41032-4

**Published:** 2019-03-13

**Authors:** Pierre Amato, Ludovic Besaury, Muriel Joly, Benjamin Penaud, Laurent Deguillaume, Anne-Marie Delort

**Affiliations:** 1 Université Clermont Auvergne, CNRS, SIGMA Clermont, ICCF, F-63000 Clermont-Ferrand, France; 2 Université Clermont Auvergne, CNRS, LaMP, F-63000 Clermont-Ferrand, France

## Abstract

Clouds constitute the uppermost layer of the biosphere. They host diverse communities whose functioning remains obscure, although biological activity potentially participates to atmospheric chemical and physical processes. In order to gain information on the metabolic functioning of microbial communities in clouds, we conducted coordinated metagenomics/metatranscriptomics profiling of cloud water microbial communities. Samples were collected from a high altitude atmospheric station in France and examined for biological content after untargeted amplification of nucleic acids. Living microorganisms, essentially bacteria, maintained transcriptional and translational activities and expressed many known complementary physiological responses intended to fight oxidants, osmotic variations and cold. These included activities of oxidant detoxification and regulation, synthesis of osmoprotectants/cryoprotectants, modifications of membranes, iron uptake. Consistently these energy-demanding processes were fueled by central metabolic routes involved in oxidative stress response and redox homeostasis management, such as pentose phosphate and glyoxylate pathways. Elevated binding and transmembrane ion transports demonstrated important interactions between cells and their cloud droplet chemical environments. In addition, polysaccharides, potentially beneficial for survival like exopolysaccharides, biosurfactants and adhesins, were synthesized. Our results support a biological influence on cloud physical and chemical processes, acting notably on the oxidant capacity, iron speciation and availability, amino-acids distribution and carbon and nitrogen fates.

## Introduction

The outdoor atmosphere harbors diverse microbial assemblages composed of bacteria, fungi and viruses (e.g.^1^) whose functioning remains largely unexplored. While the occasional presence of Human pathogens or opportunists can cause potential hazard^2,3^, in general the vast majority of airborne microbes originate from natural environments like soil or plants, with large spatial and temporal variations of biomass and biodiversity (e.g.^4,5^). Once ripped off and aerosolized from surfaces by mechanical disturbances such as those generated by wind, raindrop impacts or water bubbling^6,7^, microbial cells are transported upward by turbulent fluxes^8,9^. They remain aloft for an average of ~3 days^10^, a time long enough for being transported across oceans and continents^11–13^ until being finally deposited, eventually helped by water condensation and precipitation processes; microbial aerosols themselves can contribute to form clouds and trigger precipitation by serving as cloud condensation nuclei (CCN)^14^ and ice nuclei (IN)^15,16^.

Living airborne microorganisms may end up concretizing aerial dispersion by colonizing their new habitat^17^, provided that they survive their journey from emission to deposition. Bacterial survival is indeed naturally impaired during atmospheric transport^18,19^, but a fraction remains viable^20,21^. At high altitude, the peculiar environments offered by cloud droplets are thus regarded in some aspects as temporary microbial habitats, providing water and nutrients to airborne living cells^22–24^. In addition, the detection of low levels of heterotrophy^25^ raised questions about microbial functioning in cloud water and its potential influence on the chemical reactivity of these complex and dynamic environments^24,26^. The metabolic functioning of microbial cells in clouds is still albeit unknown, while fundamental for apprehending microbial life conditions during long distance aerial transport and their geochemical and ecological impacts.

Within the last decade, coordinated metagenomics and metatranscriptomics studies provided new insights into microbial ecosystems’ functioning and the relationships that microorganisms maintain with their environment. These were pictured in soil^27^, ocean^28,29^, human gut^30^ and elsewhere^31,32^. In the atmosphere, though, microbial gene expression and metabolic functioning remain largely unexplored, in part due to low biomass and sampling difficulties. So far, metagenomics confirmed high fungal, bacterial and viral biodiversity^33–36^, whereas targeted genomics/transcriptomics towards ribosomal genes supported earlier findings about the maintenance of metabolic activity in aerosols^37,38^, and in clouds^5^; Alpha- and Gamma-Proteobacteria in particular were highlighted. Consistently, in atmospheric chamber airborne bacteria were demonstrated to react to the presence of carbon substrate by regulating ribosomal gene expression^39^.

Here we aimed at specifying microbial activity in clouds. We performed a comparative combined metatranscriptomics/metagenomics analysis to explore their metabolic and physiological functioning, their potential interactions with cloud water chemical environment, and to examine the constraints imposed by cloud environments to living microorganisms. Cloud water samples were collected from puy de Dôme Atmospheric station (1465 m a.s.l., France), and whole metagenomes and metatranscriptomes were amplified and explored for biodiversity and biological functions. Comparative analysis highlighted a diverse biological system driven by prokaryotes. Metabolism was seemingly directed for a large part toward acclimatation to a demanding environment, including elevated oxidants and low temperatures. This gives an unprecedented picture of microbial life conditions in clouds and specifies possible biological impacts on the chemical reactivity.

## Material and Methods

### Sample collection

Cloud water was collected from the instrumental platform situated on the roof of the meteorological station at the summit of puy de Dôme Mountain (1465 m a.s.l., 45.772°N, 2.9655°E, France). Protocols similar as in Amato *et al*.^5^ were used. The volumes of water collected after sampling periods of five consecutive hours were immediately processed within the station’s microbiology facility. Subsamples for total cell counts and chemical analyses were first collected and the remaining volumes were filtered through 0.22 µm porosity (MoBio 14880-50-WF). Filters were then cut in halves, transferred into ca. 5 mL of RNA Later (Sigma-Aldrich, Saint-Louis, MO, USA) and stored at −80 °C until being further processed.

### Meteorological data, backward trajectories, cell counts and chemical analyses

Protocols similar as in Amato *et al*.^5^ were used for characterizing the samples. Briefly, meteorological data were provided by puy de Dôme’ meteorological station, backward air mass trajectories were generated using HYSPLIT^40^, ion concentrations were measured by ion chromatography (Dionex, Sunnyvale, CA, USA), and cells counts were performed by flow cytometry (BD FacsCalibur, Franklin Lakes, NJ, USA) on SYBR-Green stained samples (Molecular Probes Inc., Eugene, OR, USA).

### Ice nucleation assay

The concentration of ice nucleating particles (INP) in the cloud water samples were examined by droplet freezing assay, as in Joly *et al*.^41^. Freshly collected samples were distributed into volumes of 20 µL in 0.2 mL microtubes and exposed to decreasing temperature from 0 °C to −10 °C in a cryobath (Julabo F34-ED), and the cumulative concentration of INP at each temperature was calculated (see Joly *et al*., 2014 for details).

### Nucleic acids extraction, amplification and sequencing

DNA and RNA were extracted using MoBio PowerWater isolation kits (now Qiagen, Hilden, Germany) following manufacturer’s recommendations, from dedicated filter halves kept at −80 °C in RNA Later. We attempted to generate sequencing libraries directly from DNA extracts and cDNA extracts; these could not be validated by the quality controls due to low amounts of material (0.5–0.7 ng DNA/µL). Hence, untargeted amplification was performed: whole metagenomes (MG) and whole metatranscriptomes (MT) were amplified by multiple displacement amplification (MDA) of genomic DNA and total RNAs, from volumes of 10 µL of the corresponding extracts using REPLI-g Cell WGA & WTA kit (Qiagen).

Tropospheric clouds are among the lowest biomass environments on Earth, and sampling large volumes of cloud water within a short timeframe is still not an option, so the only alternative for accessing the nucleic acid sequences was amplification. Although MDA is known to introduce biases in the relative abundance of sequences in complex samples^42^, this provided valuable information in low biomass environments such as sediments or permafrost^43,44^. Shotgun libraries were generated using Nextera XT DNA Sample Preparation Kit. These were controlled for quality on Agilent High Sensitivity microarray, and mixed in an equimolar pool for sequencing (2 × 300 bp paired-end Illumina MiSeq; Genoscreen, Lille, France). Experimental blank controls led to exploitable library only for MT. The corresponding sequence files were deposited to NCBI’s Sequence Read Archive (BioProject ID PRJEB25763) with the sample accession numbers ERS2351639 to ERS2351645.

### Sequence processing and data analysis

All sequences were processed using free software and custom Perl scripts, using the regional calcul center Mesocentre Clermont Auvergne (in general 32 CPUs with 128 Go of RAM were used here), and locally under Linux Ubuntu operated computers. Sequences were first quality controlled (FastQC version 0.11.3; Babraham Bioinformatics) and trimmed for removing low quality ends, sequences < 40 bp or containing ambiguous bases using PRINSEQ-LITE^45^. Mate pairs were assembled using PANDAseq Assembler version 2.8^46^; final sequence length was ~300 bp. Potential contaminant sequences were removed by alignment against the experimental control using BWA-MEM^47^ and SAMtools^48^.

Taxonomic annotations of prokaryotes and eukaryotes were obtained using BLASTN^49^ against the ribosomal database SILVA 119.1 SSURef Nr99^50^; the best hits with an alignment e-value < 0.01 were recovered. Chloroplasts and mitochondria sequences were manually removed from the analysis.

Functional annotations were performed using BLASTX^49^ against the protein database UniprotKB^51^, restricted to cellular organisms (Archaea, Bacteria and Eukaryotes) and viruses. The best hits with an alignment e-value <10^−4^ were collected. Data were then analyzed using gene ontologies (GO IDs terms defined by the Gene Ontology Consortium)^52–54^ associated with UniprotKB identifiers and using notably the following informatics tools and databases: Protein Information Resource PIR^55^, REVIGO^56^, GOSlimViewer^57^, AmiGO^53^, and KEGG^58–60^. In total, 2670 to 3373 unique GO terms were found in each cloud water MG or MT dataset.

For comparative analysis, other sequence files were collected from literature studies selected for including both metagenomes and metatranscriptomes (Table S1), and these were reprocessed similarly as our data using our bioinformatic pipeline. When necessary, data were rarefied to ~400,000 sequences to remain consistent between datasets. Rarefaction may have decreased the sensitivity of the intercomparison, but this is acceptable in the frame of our descriptive study looking for large differences originating from multiple features (GO terms), rather than a direct comparison of the abundance of specific sequences^61^. GO terms represented by ≥0.05% of the sequences in at least one MT dataset were considered (823 GO terms in total; Table S2). Among those, 724 occured in cloud MT and 764 in MG, and 699 were present in both MT and the corresponding MG: 317 of these related to a Molecular Function (MF), 313 to a Biological Process (BP), and 69 to a Cellular Component (CC). Their corresponding rank-abundance in MT and relative abundance respect to MG are shown in Figs S1 and S2. The GO were finally manually classified according to functional categories, and their relative level of expression was assessed through their relative contribution in MT (i.e. %MT), and after normalization to their relative representation in MG (i.e. %MT/%MG). This approach is commonly used in metatranscriptomics studies^30,62^, and it has the advantage to overcome problems related to unknown gene copy numbers.

## Results and Discussion

The main meteorological, chemical and biological characteristics of the cloud water samples are presented in Table 1.

**Table 1:** Main characteristics of the cloud water samples.

Sample Identifier	Cloud 20141117-1	Cloud 20141117-2	Cloud 20141117-3
Date	17 Nov 2014	17–18 Nov 2014	18 Nov 2014
Local sampling time	3:22 pm–8:22 pm	8:23 pm–1:23 am	3:50 am–8:50 am
Volume of sample processed (mL)	160	120	90
Total cell concentration in cloud water (N mL^−1^)^(a)^	(2.8 ± 0.7) × 10^3^	(2.8 ± 0.7) × 10^3^	(2.7 ± 0.5) × 10^3^
Inferred total cell concentration in cloud air (N m^−3^)^(a) (b)^	(8.3 ± 2.2) × 10^2^	(6.3 ± 1.6) × 10^2^	(4.5 ± 0.9) × 10^2^
Ambient temperature during sampling (°C)	0.7	0.6	0.4
Wind speed (m s^−1^)	7.4	9.9	10.3
pH	5.4	5.0	4.9
**Ion concentrations (µM)** ^**(a)**^			
SO_4_^2−^	5.7 ± 1.9	7.0 ± 0.4	3.0 ± 1.1
NO_3_^−^	4.5 ± 0.15	9.2 ± 0.7	8.6 ± 0.1
Cl^−^	0.7 ± 0.1	1.0 ± 0.4	1.0 ± 0.1
NH_4_^+^	15.4 ± 3.6	20.6 ± 2.0	50.3 ± 3.5
Na^+^	4.8 ± 5.5	11.3 ± 14.2	13.7 ± 2.0

^a^Mean of triplicate measurements ± standard error.

^b^Considering sampling at the constant air flow rate of 108 m^3^ of air h^−1^.

Samples were collected at ambient temperatures close to freezing, under moderate to strong winds with bursts up to 14 m/s; liquid water content varied between 0.16 and 0.47 mL m^−3^. Air masses originated from West (Atlantic Ocean; Fig. S3), and the chemical signature of water samples (pH, dissolved ion concentrations) was consistently typical of “Marine” type clouds at this site^63^. Total cell concentration was ~2.8 × 10^3^ cells mL^−1^ of cloud water, i.e. ~4 × 10^2^ to ~9 × 10^2^ cells m^−3^ of cloud air; this is at the very lower end of microbial biomass usually observed^64^ confirming the background-type situation (i.e. low influence of Human activities).

### Community composition

**Figure 1:**
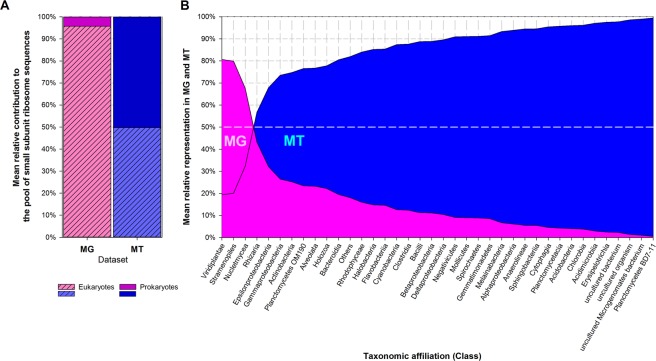
(**A**) Mean relative contribution of eukaryotic (dashed) and prokaryotic (clear) taxa to the pool of identified SSU rRNA gene sequences in metagenomes (MG, pink) and in metatranscriptomes (MT, blue). (**B**) Mean relative distribution between MG and MT of the dominant taxonomic classes, ordered by increasing representation in MT.

Biodiversity was assessed from ribosomal sequences; this does not necessarily represent cell number distribution. Eukaryotes largely dominated in metagenomes (MG) (~95% of the ribosome sequences; Fig. 1), as expected from their much higher ribosome gene copy number than prokaryotes: up to thousands copies in eukaryotes *versus* ~1–15 copies in bacteria^65,66^. Most were affiliated with Nucletmycea (equiv. Holomycota), Viridiplantae and Stramenopiles-Alveolata-Rhizaria (SAR), i.e. fungi, plants/algaes and planctonic unicellular organisms. Prokaryotes consisted essentially of bacteria, dominated by Proteobacteria, over Firmicutes, Bacteroidetes, Acidobacteria, Actinobacteria, and Chloroflexi as often in atmospheric samples^67^. Conversely, prokaryotes sequences were much more abundant in metatranscriptomes (MT) (50.2% ± 10.9% of the ribosome sequences) than they were in MG. Their mean relative representation in MT, with respect to MG, was thus ~20 times greater than that of eukaryotes (5.7 versus 0.26, respectively), suggesting probable higher protein synthesis and metabolic activity^27^; this emphasized bacteria in particular as the active microbiota of clouds. Based on this ratio, the most active bacterial taxa comprised notably groups related with water environments, like Planctomycetes, Chlorobia and Cyanobacteria, along with bacteria shown previously to maintain metabolic activity in clouds, such as Alpha and BetaProteobacteria^5^.

Apart from ribosomal sequences, a number of functional genes indicated the presence of viruses. Viruses are frequently reported among aerosols, including potential pathogens^3,68^. We observed here the presence of dsDNA and ssRNA viruses and retroviruses, known to infect with more or less specificity arthropodes (Baculoviridae, Poxviridae, Picornavirales), plants (Ourmiavirus, Tymovirales), animals and Humans (Herpesviridae, Flaviviridae, Mononegavirales), aquatic organisms like fish and amphibians (Iridoviridae, Phycodnaviridae) or fungi (Hypoviridae).

### Functional analysis

**Figure 2:**
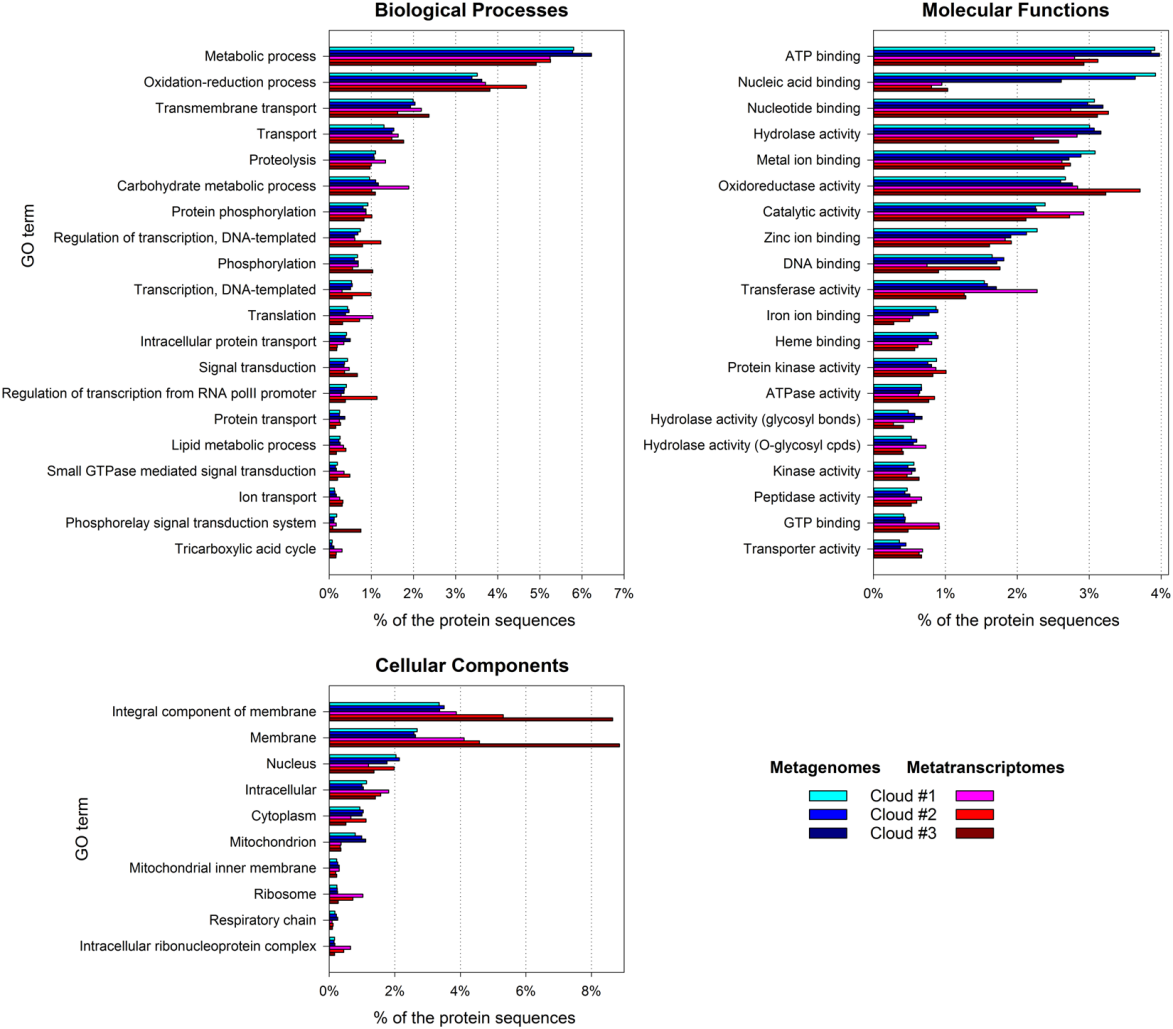
Main gene ontology terms related with Biological Processes, Molecular Functions and Cellular Components in cloud metatranscriptomes (blue), and in the corresponding metagenomes (red).

In order to examine community functioning, we explored the gene ontologies (GO) associated with identified protein sequences. The relative distribution of GO terms among MG and MT datasets was variable, thus highlighting metabolic requirements within community’s capabilities. Overall, metabolic processes oriented toward energy production (catabolism) and transports dominated (Fig. 2).

Accordingly, among cellular components, membranes and ribosomes were highly represented in MT, relatively to MG. Meanwhile, cellular nucleus and mitochondria tended to be underrepresented in MT compared with MG, supporting prokaryotes as the active microorganisms.

In the absence of reference, the information provided *per se* by metranscriptomes for examining the microbial life conditions in a particular environment is limited. Hence, since our study of cloud water microbial communities has no counterpart yet in the atmosphere, and with the objective to reveal the specificities and constraints imposed by clouds to living microorganisms, we assessed MT in regard to the corresponding MG^29^, and we based our interpretation on a comparative analysis between clouds and other environments, obtained from reprocessed literature data selected for including both metagenomes and metatranscriptomes on same samples (see Table S1**)**.

#### Comparative metatranscriptomics specifies harsh microbial lifestyle in clouds, driven by oxidants and low temperatures

**Figure 3:**
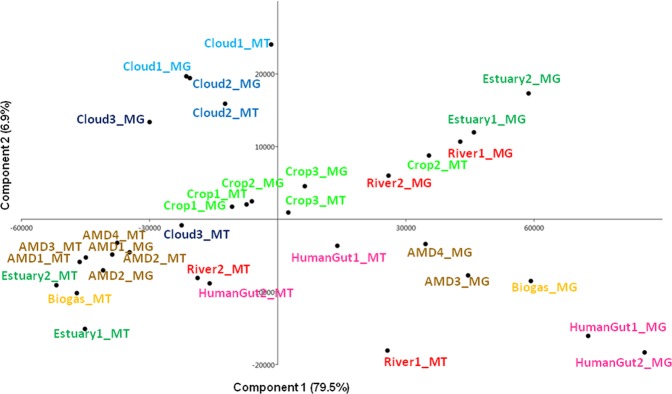
Principal component analysis representation of metagenomes (MG) and metatranscriptomes (MT) datasets in clouds (this study) and in other environments (from litterature data, see Table S1 for references and details), based on sequence distribution among the GO terms obtained from identified protein sequences.

The biological processes exhibited by the cloud microbiota were compared to those observed in other environments using similar approaches and available in literature (see SI for details): crop rhizosphere (n = 3)^69^, river (Amazon) (n = 2)^31^, estuary (Columbia river) (n = 2)^70^, biogas fermenter (n = 1)^71^, Human gut (n = 2)^30^, and acid mine drainage (AMD; n = 4)^32^. Overall, clouds were distinct from the other environments assessed (principal component analysis PCA; Fig. 3), with MT closely related to corresponding MG. In the case of Cloud 3, MG and MT were clearly separated, and MT resembled other known environments such as crop and river. It is not possible to conclude here on the reason(s) for the apparent distinct biological functioning of Cloud 3; it may be related to changes in air mass characteristics (chemical composition; see Table 1), or to other environmental feature like sunrise. Such variations on such a short period of time illustrates the great short-term variability of biodiversity existing in the atmosphere^72^. Moreover, large short-term variations of the expression of biological functions are regularly reported in other environments like seawater^73^.

**Figure 4:**
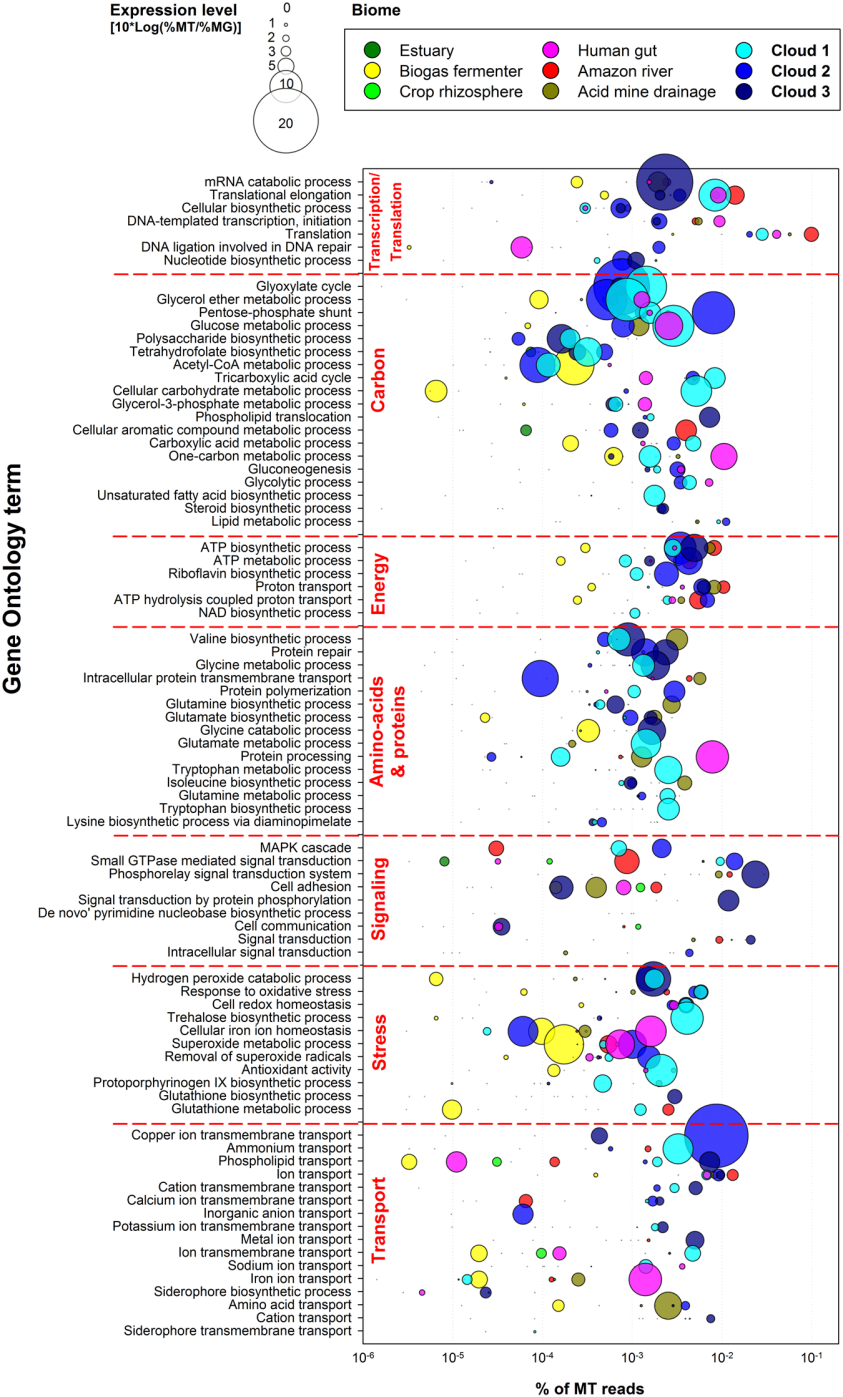
Biological processes expressed by cloud communities, compared with other environments (see Table S1 for details and references). Bubble size depicts function’s expression level; as expressed it is equal to 0 for similar representation in MT as in MG, and >0 for greater representation in MT, so only overexpressed functions are visualized. See Table S2 for the complete GO term list and the number of corresponding sequences in each dataset.

The main results of the functional analysis are presented in Fig. 4 and the corresponding data are shown in Table S2; these are to be consulted throughout this section. More details are presented in SI Figures, which are referenced when appropriate. These unique datasets allowed us to draw an unprecedented picture of cloud microbiota’s functioning and of its relationships with its temporary cloud water habitat.

Our analysis revealed the expression of biological functions reflecting a challenging environment, largely oriented toward the response to a demanding environment as one can figure: oxidant, cold, and subjected to osmotic variations. These thus seemingly drove many aspects of cell functioning, as depicted here, from carbon metabolism to transports. Living cells indeed deployed multiple processes largely involved in the maintenance of homeostasis and in the response to oxidative stress (Fig. S4). Free radical (superoxide) and oxidant (hydrogen peroxyde) detoxification processes themselves strongly solicited cells through enzymes such as catalase (E.C. 1.11.1.6; GO:0004096), superoxide dismutase (E.C. 1.15.1.1; GO:0004784), peroxiredoxin (E.C. 1.11.1.15; GO:0051920) and peroxidase (E.C. 3.5.2.6; GO:0004601), and antioxidant compound synthesis (glutathione; GO:0006750).

Additionally, transition metal (copper and iron) transport was notable (Fig. S5); these participate to various oxidant detoxification enzymatic reactions^74,75^. Noteworthy, siderophore synthesis and transport processes were detected; these are high affinity iron complexing compounds released by cells in their surrounding when deficient, notably by numerous *Pseudomonas* species isolated from clouds^76^.

The implication of membranes, ribosomes and protein complexes is confirmed in the comparative analysis (Fig. S6). Elevated transmembrane transports and binding activities suggested important interactions between cells and their direct surroundings. The respective phosphorylation level stimuli and signals transduction systems from the environment to cell’s inside, guenine cell’ sensing “organs”^77^, indicated that mostly bacteria, but not only, were attempting to respond/acclimate to their environment (MAPK cascades in eukaryotes; GO:0000165^78^; and phosphorelay systems in bacteria; GO:0000155 and GO:0000160^79^) (Fig. S4). The high calcium transport activity observed was potentially acting in signal transduction^80^.

Despite temperatures close to freezing during sampling, translational activity, i.e. protein synthesis was maintained (Fig. S7). This is consistent with the reported capacity of cloud microbial communities to uptake ^14^C labelled leucine at 0 °C^25^. In all likelihood ammonium, abundant in the samples (Table S1), was taken up from clouds water and fed amino-acid metabolism with nitrogen (Fig. S5)^81^. This was oriented toward valine, glycine, glutamate and glutamine metabolisms; in addition of permitting new proteins synthesis implied by metabolic arrangements, glycine and glutamate are constituents of gluthathione, the main intracellular redox regulator. Glutamine and glutamate have a central role in nitrogen and amino-acid metabolisms, which they connect with central metabolism (TCA or glyoxylate cycles). Metabolomic profiling of the bacterium *P. syringae* isolated from clouds highlighted increased concentration of these amino-acids in cells exposed to cold^82^.

The synthesis of compatible solutes like trehalose (GO:0005992) or glycine betaine, from glycine metabolism, allowed cells to endure cold and osmotic variations^83,84^. Functions of lipid metabolism and transport indicated other responses to cold: membrane synthesis and modifications. High activities of glycerol ether, steroid, phospholipid and unsaturated fatty acid metabolisms were indeed occurring (Fig. S8), with enzymes such as stearoyl-CoA desaturase (E.C.1.14.19.1; GO:0004768), along with branched-chain amino-acid (BCAA) biosynthetic processes (GO:0009082) and phospholipid transport (Fig. S5). Adjustments (increase) of fatty acids unsaturation level in membrane for maintaining fluidity is a well known acclimatation to cold in Gram-negative bacteria^85^, while Gram-positive bacteria rather adjust the branching of their branched-chain fatty acids, involving BCAA biosynthesis^86^.

Laboratory investigations of cloud microbial isolates indicated that H_2_O_2_, at its cloud water concentration of < ~0.1 mM, was not altering microbial survival^87^. When exposed to cold, bacteria such as *Pseudomonas syringae* exhibit many simultaneous metabolic regulations similar as those observed here, and imparting increased tolerance to changing environmental conditions: metabolism rerouting, compatible solutes and antioxidants synthesis, membrane modifications or again increased biochemical energy production^82^.

These defenses and acclimatation mechanisms were associated with marked activities of energy transducer and redox cofactor synthesis (riboflavin, NAD and ATP, through proton translocation; Fig. S9). These attested of a substantial demand of energy for feeding the biosynthetic pathways and physiological responses ongoing here^88^. Accordingly, the cell machineries were likely essentially fueled by metabolic pathways including glyoxylate, tricarboxylic acids (TCA) and pentose phosphate cycles (Fig. S8). These are known to greatly contribute to cell homeostasis and fight oxidants, consistently with the other functions observed. In clouds, indeed, ATP concentration was reported to vary in relation with that of H_2_O_2_ suggesting metabolic regulations linked with oxidative stress response^89^.

TCA cycle is a central pathway common to all aerobic organisms and dedicated to the production of energy and reducing power from acetyl moieties (acetyl-CoA). This corresponds to the final oxydation steps of carbohydrates, lipids and amino-acids into CO_2_. Numerous key enzymes of the TCA cycle were found overexpressed here, including succinate dehydrogenase, citrate synthase, phosphoenolpyruvate carboxykinase (ATP), and pyruvate dehydrogenase (Fig. S10). The glyoxylate cycle is a shortened alternative to the TCA cycle sharing several steps with it, but avoiding carbon dissimilation for allowing biomass production from C_2_ substrates like acetate^90^. Glyoxylate pathway is notably used for fatty acid synthesis, and it was found to be connected with the response to cold and oxidative stress in several Alpha- and Gamma-proteobacteria (*Caulobacter*, *Colwellia*, *Pseudoalteromonas*, *Pseudomonas*, *Psychrobacter*, *Rhizobium* and others)^91–94^. Several enzymes specific of this pathway were detected at high level in MT, including malate synthase and isocitrate lyase (Fig. S11). The pentose phosphate shunt is a major pathway involved in the regulation of cell redox homeostasis, in addition to having a central role in the biosynthesis of amino acids and RNA precursors. This leads to the production and regeneration of NADPH^95,96^, the redox cofactor required for recycling the main cell’s antioxidant machinery intermediate: gluthathione. Related enzymes such as ribose phosphate diphosphokinase, fructose-bisphosphate aldolase phosphogluconate dehydrogenase (NADP + dependent, decarboxylating) and again fructose 1,6-bisphosphate 1-phosphatase (Fig. S12) were found overexpressed, along with, accordingly, NADP binding activity (GO:0050661).

Other remarkable carbon metabolic pathways included glucose metabolic processes and polysaccharide synthesis (Fig. S8). This was possibly linked with the synthesis of exopolysaccharides (EPS), known to protect cells from environmental variations and dessication^97^ and emphasized in cloud bacteria isolates^98^, as were biosurfactants^99^, and/or of adhesins, compounds involved in cell adhesion and aggregation (GO:0007155)^100^. The production of such compounds could be beneficial for the survival of airborne living cells^101,102^. The carbon routes detected overexpressed in clouds based on the identification of specific enzymes are summarized in Fig. S13.

Finally, this analysis can give hints about the potential carbon substrates uptaken by microorganisms in cloud water. Although the exact substrate(s) remain(s) undetermined here, biological processes involving one-carbon compounds (GO:0006730) were occuring (Fig. S8). Tetrahydrofolate (THF) synthesis (GO:0046654) notably was high; it acts as a C_1_ metabolism cofactor in plants and microorganisms, allowing methylotrophy for biosynthetic processes^103^. In the atmosphere, C_1_ compounds are among the most abundant carbon compounds (e.g.^63^); they are end-products of organic compounds oxidation before their complete mineralization into CO_2_. Laboratory incubation of cloud water previously demonstrated a biological role in their degradation^26^, and viable facultative methylotrophic bacteria like *Pseudomonas* spp. or *Methylobacterium* spp. are frequently detected in atmospheric samples^5,22,24^.

#### Potential implications for atmospheric chemical and physical processes

Clouds are highly dynamic environments, with extremely complex chemistry and microphysics that have huge impacts on atmosphere and climate, yet far from being well known and understood^104–106^. The current view in cloud water chemistry is that this is driven by free radicals, generated by sources including direct photolysis of hydrogen peroxide (H_2_O_2_), its dissolution from the surrounding gas phase, and its reactivity with transition metals and nitrate ions^107^.

**Figure 5:**
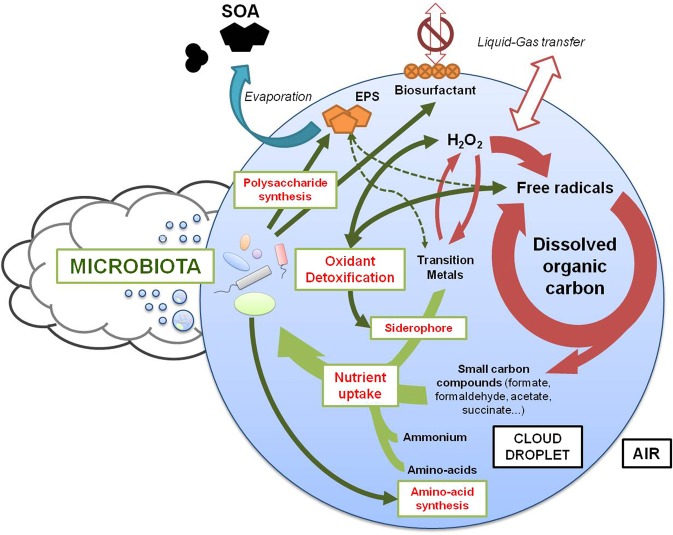
Schematic summary of the main probable impacts of microbial activity on cloud processes, based on coordinated metagenomics/metatranscriptomics. Biological processes and their targets are indicated by green arrows, while red arrows indicate abiotic processes. EPS: Exopolysaccharide; SOA: Secondary organic aerosol.

Biological particles and activity were regularly proposed as probably involved in processes such as cloud formation^14^, precipitation triggering^108^ and chemical reactivity^26^. Figure 5 illustrates the biological processes identified here as taking place in clouds and potentially affecting chemistry and physics. First, our results support earlier suggestions based on laboratory observations that biological activity may regulate, at least in part, the oxidant and radical concentrations in clouds^26^ and contribute to the degradation of carbon compounds including, but not limited to, formate, formaldehyde, acetate and succinate^109–111^. In addition, metals were shown to be largely complexed by undefined organic compounds in atmospheric water, thus limiting their chemical availability^112^. Such strong complexants as siderophores, which the synthesis is evidenced here, are likely involved^113^.

Beside this, the abundance and distribution of amino-acids in cloud water were examined earlier at the same sampling site^114^. Total amino-acids amounted ~3 µM (~9% of the dissolved OC), and the distribution of the 16 amino-acids detected and quantified was not equimolar, but it was dominated by tryptophane, isoleucine, and phenylalanine. Here we observed overexpression of tryptophane and isoleucine biosynthesic pathways, indicating that cloudborne microorganisms themselves could be responsible for the uneven distribution of amino-acids observed.

The activity of polysaccharide synthesis possibly corresponded to biosurfactants and exopolysaccharides (EPS). The former can influence cloud formation and exchanges between liquid and gas phases^115^, whereas EPS scavenge oxidants^116^, complex metals^117^, and represent potential secondary organic aerosols once cloud dissipates^97^.

Finally, ice nucleation (IN), in particular at temperatures >−10 °C, i.e. catalyzed by biological entities, is a topic of interest in atmospheric sciences as this influences precipitation^118^. There is no doubt on the presence of low numbers of ice nucleating bacteria in clouds^119^. Here, IN assays on the samples demonstrated freezing between −6 °C and −7 °C, with up to >200 IN particles mL^−1^ of water at −8 °C (Fig. S14). This is relatively high compared with previous observations and totally compatible with bacterial IN activity^41^; however, no known gene coding for IN activity was observed; detecting specific signature of such rare microbial phenotypes or traits in nucleic acids requires targeted methods^120^.

In conclusion, we provided here the first molecular picture of microbial living conditions in the top layer of the biosphere constituted by clouds. As samples were collected during the night, we did not detect photosynthetic activity, but the presence of diverse phototrophic organisms indicates that this potentially occurs. A different picture could have been drawn under different environmental configuration like during the day, in the case of pollution events from anthropogenic or natural emissions, under warmer/colder conditions etc, which then remain to be explored. Numerous aspects led us to affirm that cloud droplets are demanding habitats for living cells, challenged to respond or acclimate to oxidative stress, low temperatures and osmotic variations. Thus, specific central metabolic routes directed toward the management of oxidants were preferred for producing the energy required for homeostatis, synthesis of protective compounds and physiological rearrangements. In microbial evolution, atmospheric dispersal could have promoted the maintenance and diversification of such phenotypes by exerting strong selection pressure on inapt individuals, while allowing others to spread over the planet. Furthermore, oxidants are considered as the main drivers of atmospheric chemistry. Their regulation in cloud water by the multiple ways deployed by living microorganisms clearly positions microbial cells as central actors, to some extent, of cloud chemical reactivity. The results also suggest biomass and biochemical energy production from substrates including small carbon compounds (C1 and C2, succinate), and ammonium as the source of nitrogen.

Our untargeted metatranscriptomic approach gave many insights into the functioning of microbial cells within cloud droplets, their physiological traits and potential impacts. This specified biological functions of interest, and this should help identifying specific target genes for futures investigations. The low biomass imposed a step of DNA amplification by MDA. This necessarily distorted to some extent the view of the actual nucleic acid content of the samples. Significant progress to a better evaluation of biological functions expression levels will consist of absolute quantifications of genes and transcripts, i.e. without amplification step. Furthermore, as gene expression cannot be directly related with any quantification of activity, activity measurements remain necessary to determine the actual biological imprint on clouds, and in order to take into account posttranscriptional regulations, potentially caused by environmental factors.

## Supplementary information

Supplementary material

Table S2

## Data Availability

The sequence filesgenerated in this study were deposited to NCBI’s Sequence Read Archive (BioProject ID PRJEB25763) with the sample accession numbers ERS2351639 to ERS2351645.
