# Supplementary material for: Metatranscriptomic exploration of microbial functioning in clouds
Source: Sci Rep. 2019 Mar 13;9:4383. doi: 10.1038/s41598-019-41032-4 (PMC6416334; doi:10.1038/s41598-019-41032-4)
Supplement: Supplementary file 1 — Supplementary material [file 41598_2019_41032_MOESM1_ESM.pdf]

Supplementary material to: Metatranscriptomics exploration of microbial functioning in clouds

Pierre Amato[1*], Ludovic Besaury[1], Muriel Joly[1], Benjamin Penaud[1], Laurent Deguillaume[2], and Anne-Marie Delort[1]

(1) Université Clermont Auvergne, CNRS, SIGMA Clermont, ICCF, F-63000 CLERMONT-FERRAND, FRANCE

(2) Université Clermont Auvergne, CNRS, LaMP, F-63000 CLERMONT-FERRAND, FRANCE

Phone: +33(0)4 73 40 52 84. Fax: +33(0)4 73 40 77 17. E-mail: pierre.amato@uca.fr

*Corresponding author

Table S1: Detailed information on the MG and MT datasets used for comparative metatranscriptomics approach, including literature data.

Environment	Bioproject A.N.	Environment	Sequence File A.N.	Type of Dataset	Reference	Number of sequences analyzed	% of reads with hit against UniprotKB database
**Estuary**	**PRJEB9136**	Columbia River - Salinity 0	ERX943715	MG	**Fortunato and Crump, 2015** [6]	421213	55.9%
		Columbia River - Salinity 0	ERX943716	MT		455354	77.0%
		Coastal Ocean - Salinity 33	ERX943719	MG		376185	69.4%
		Coastal Ocean - Salinity 33	ERX943720	MT		446985	69.1%
**Biogas**	**PRJEB8813**	Biogas fermenter	ERX923318	MG	**Bremges et al., 2015** [7]	413469	67.5%
		Biogas fermenter	ERX923323	MT		375565	81.5%
**Human gut**	**PRJEB22387**	Human gut (EtOH extraction) #1	SRX247397	MG	**Franzosa et al., 2014** [8]	385453	81.1%
		Human gut (EtOH extraction) #1	SRX247333	MT		395596	55.5%
		Human gut (EtOH extraction) #2	SRX247398	MG		416594	82.3%
		Human gut (EtOH extraction) #2	SRX247342	MT		393276	34.6%
**Crop**	**PRJNA254830**	Rhizosphere of biofuel crop #1	SRX648965	MG	**Guo et al., 2016** [9]	388018	33.0%
		Rhizosphere of biofuel crop #1	SRX648967	MT		469543	48.9%
		Rhizosphere of biofuel crop #2	SRX648972	MG		382077	31.0%
		Rhizosphere of biofuel crop #2	SRX648975	MT		330992	47.7%
		Rhizosphere of biofuel crop #3	SRX648978	MG		360845	35.9%
		Rhizosphere of biofuel crop #3	SRX648981	MT		402817	39.8%
**River**	**PRJNA237344**	Amazon river plume #1	SRX495672	MG	**Satinsky et al., 2014** [10]	359773	60.9%
		Amazon river plume #1	SRX500849	MT		427608	41.7%
		Amazone river plume #2	SRX866630	MG		392381	53.2%
		Amazone river plume #2	SRX861660	MT		331442	36.5%
**Acid mine drainage**	**mgp9669**	Acid mine drainage #1	mgm4568582.3	MG	**Chen et al., 2015** [11]	401040	15.0%
		Acid mine drainage #1	mgm4568577.3	MT		50137	90.5%
		Acid mine drainage #2	mgm4568583.3	MG		393212	13.9%
		Acid mine drainage #2	mgm4568579.3	MT		61834	93.0%
		Acid mine drainage #3	mgm4568585.3	MG		399581	80.6%
		Acid mine drainage #3	mgm4568580.3	MT		62018	93.5%
		Acid mine drainage #4	mgm4568661.3	MG		398479	70.4%
		Acid mine drainage #4	mgm4568581.3	MT		58557	92.3%
**Cloud (this study)**	**PRJEB25763**	Cloud water #1	ERX2505538	MG	**This study**	552592	34.5%
		Cloud water #1	ERX2505541	MT		387585	53.4%
		Cloud water #2	ERX2505539	MG		593822	32.2%
		Cloud water #2	ERX2505542	MT		285082	52.0%
		Cloud water #3	ERX2505540	MG		412245	31.1%
		Cloud water #3	ERX2505543	MT		163421	52.5%

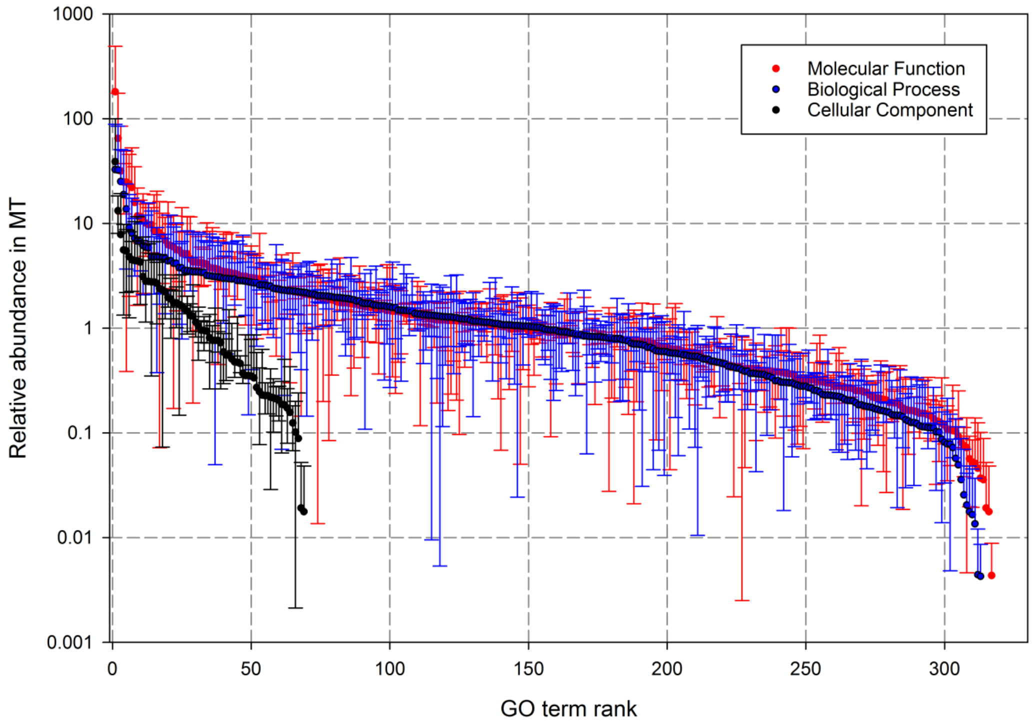

Figure S1: Rank-abundance plots of the GO terms related to Molecular Function, Biological Process and Cellular Component, expressed as the mean of their relative abundance in MT normalized to their relative abundance in MG. Errors bars are standard deviations from the mean (n=3).

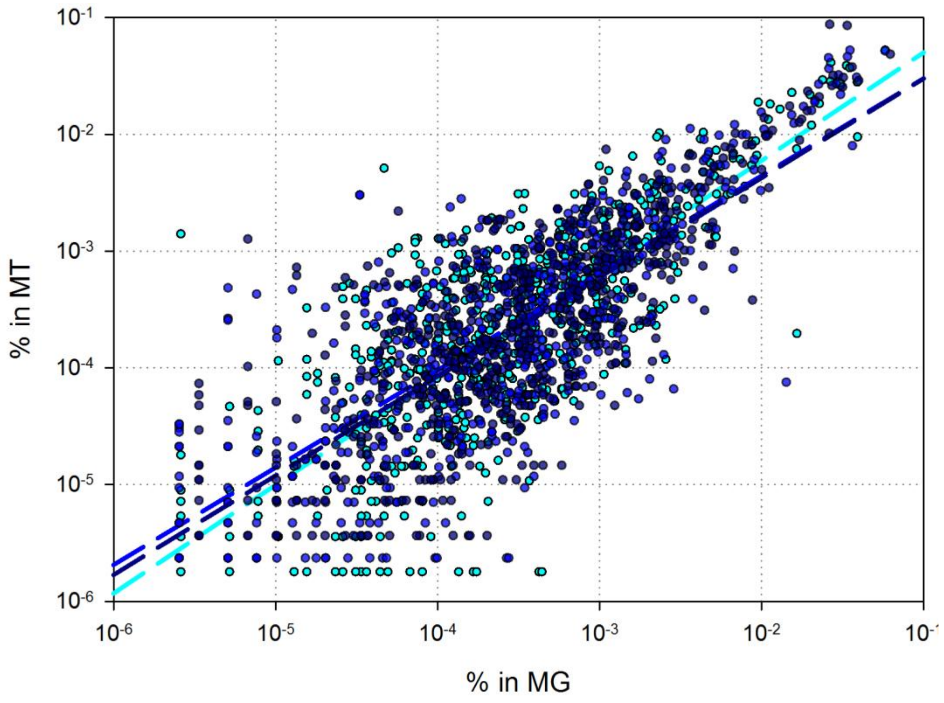

Figure S2: Relative representation of the 823 GO terms considered in the 3 cloud metatranscriptomes and in the corresponding metagenomes (in cyan, blue and dark blue, respectively). The linear fit curves have slopes of 0.894 ±0.013 (r^2 = 0.843; n=654), 0.977 ±0.014 (r^2 = 0.839, n=649) and 0.694 ±0.024 (r^2 = 0.694, n=635), respectively.

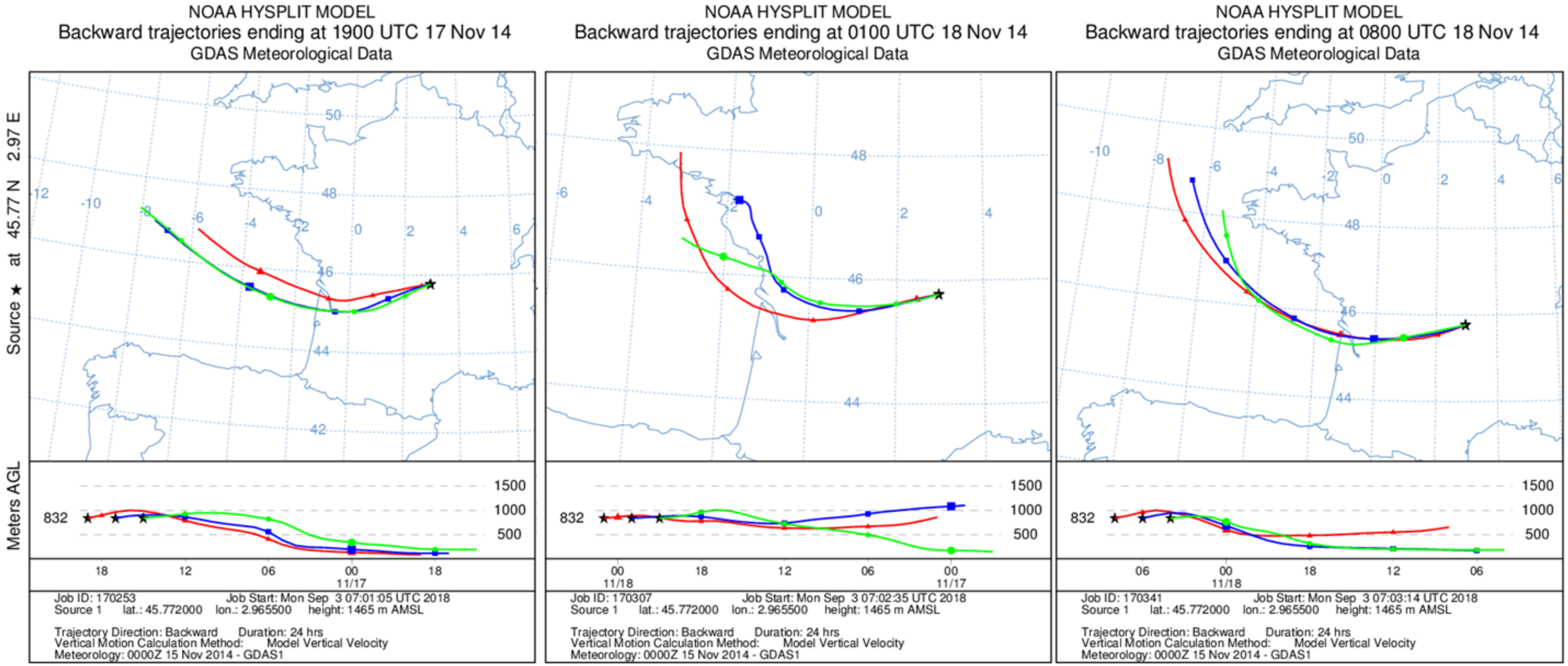

Figure S3: Twenty-four hours backward trajectory plots of the air masses arriving at puy de Dôme' summit on the sampling dates and times (NOAA HYPLIT model; [1]).

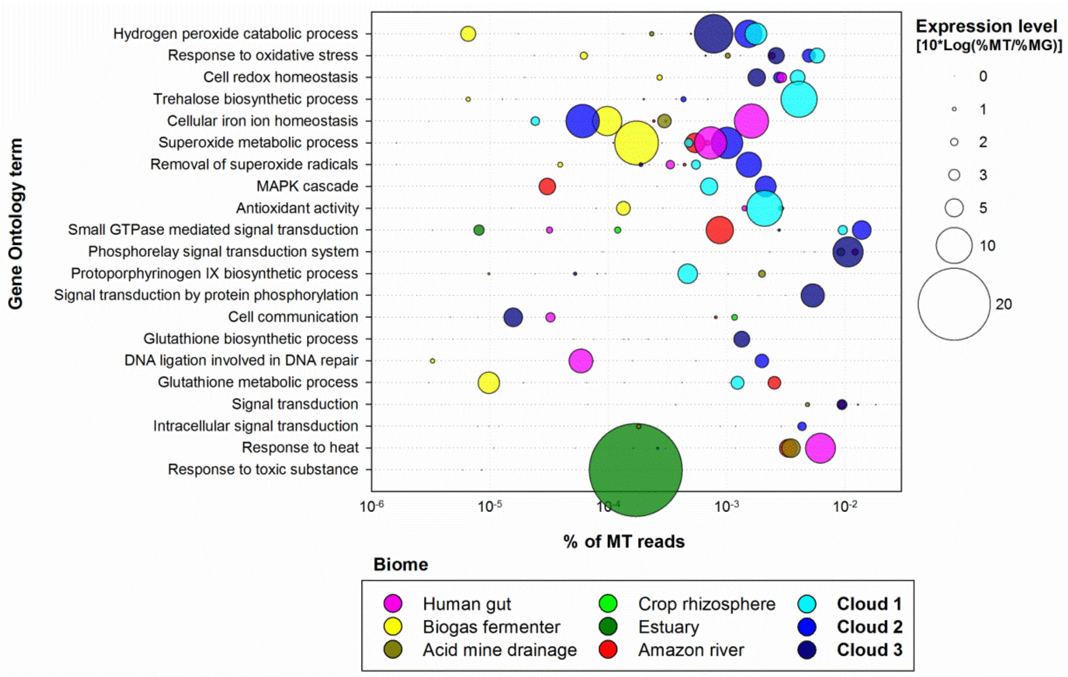

Figure S4: Biological processes related with **stress response and signaling** in clouds, compared with other environments, ordered by decreasing summed expression level in clouds (cumulated bubble size). The expression level (bubble size) depicts the relative importance of the corresponding GO term in the MT dataset(s) after normalization to MG; as expressed this is equal to 0 for similar representation in MT as in MG, and >0 for greater representation in MT, so only overexpressed functions are visualized.

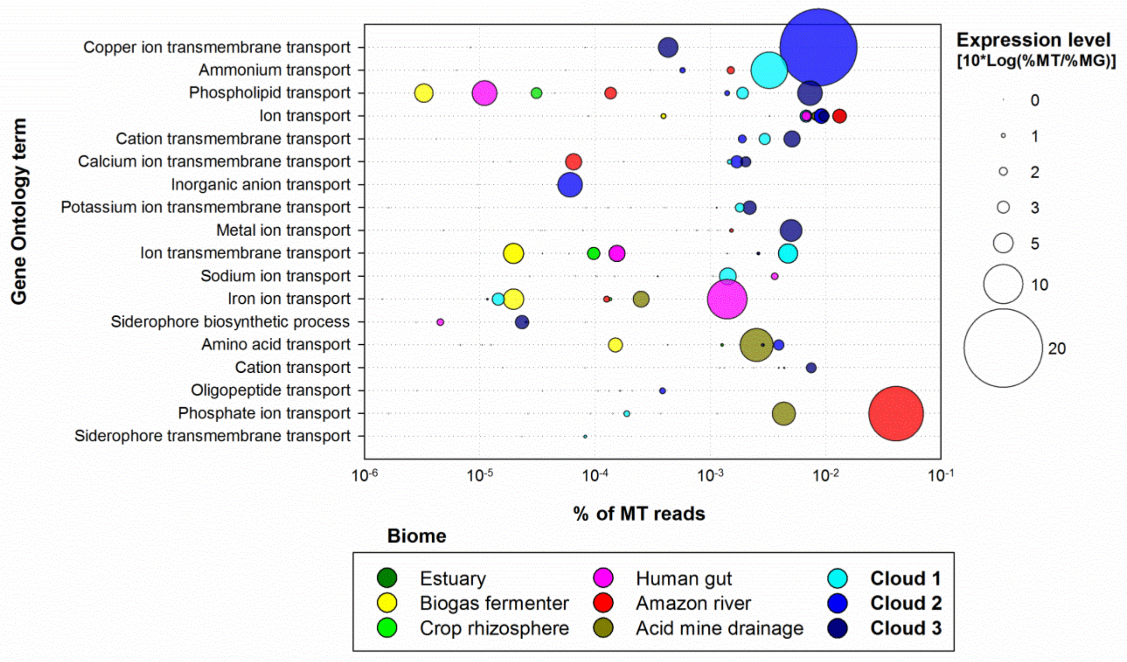

Figure S5: Biological processes related with **transports** in cloud communities, compared with other environments, ordered by their summed expression level in clouds (cumulated bubble size). See legend of **Figure S4** for further details.

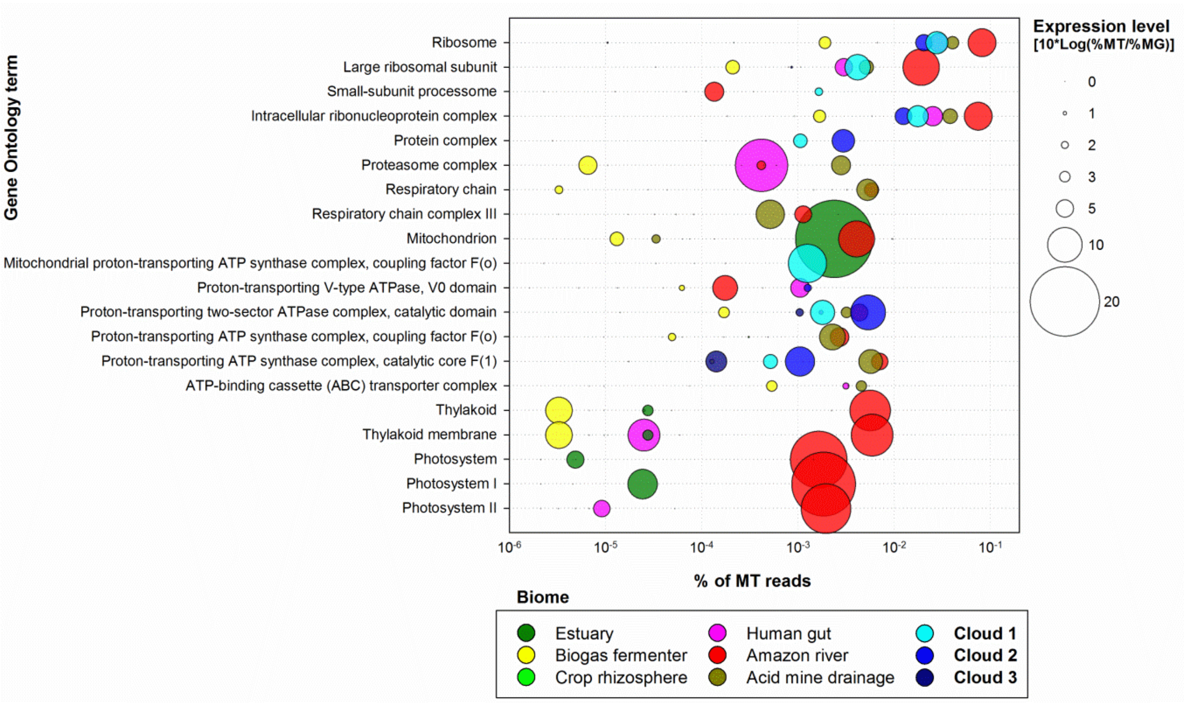

Figure S6: Main **cellular components** involved in the functionality of cloud microorganisms and in other environments, ordered by decreasing summed expression level in clouds (cumulated bubble size). See legend of **Figure S4** for further details.

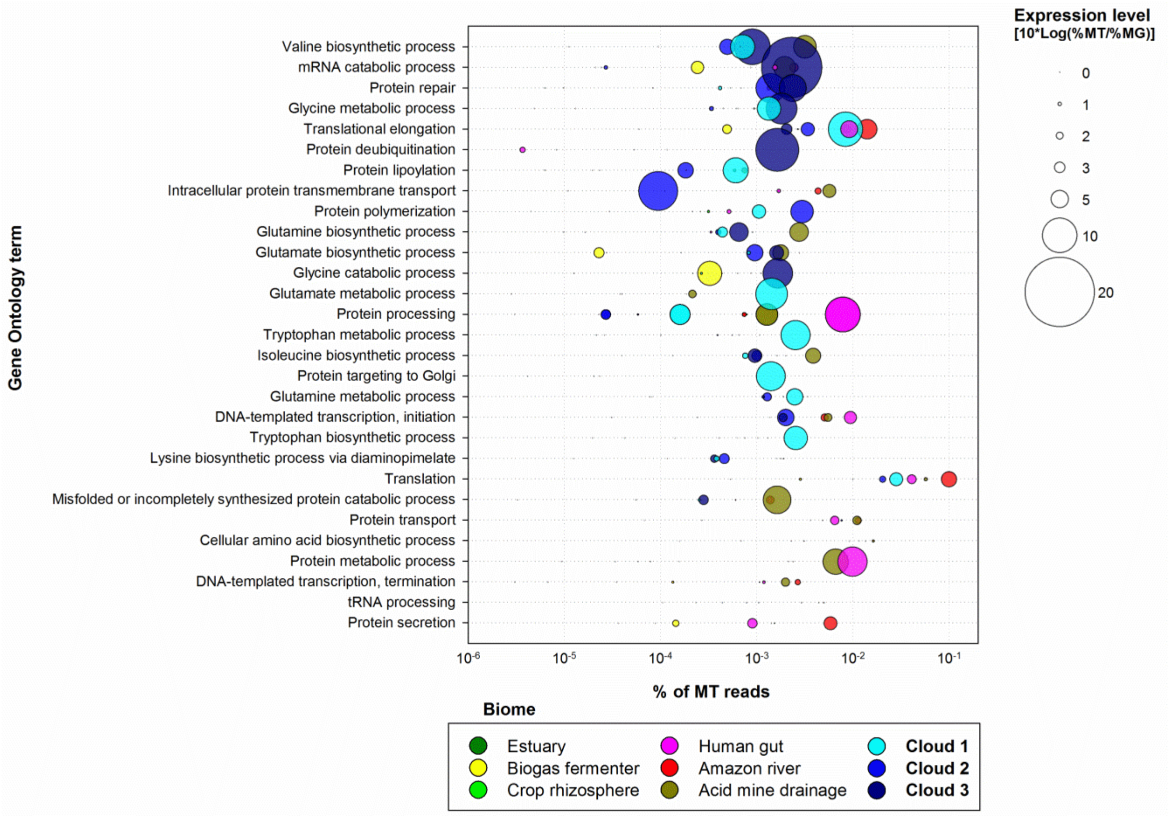

Figure S7: Biological processes related with **amino acids and protein metabolisms** in clouds, compared with other environments, ordered by their summed expression level in clouds (cumulated bubble size). See legend of **Figure S4** for further details.

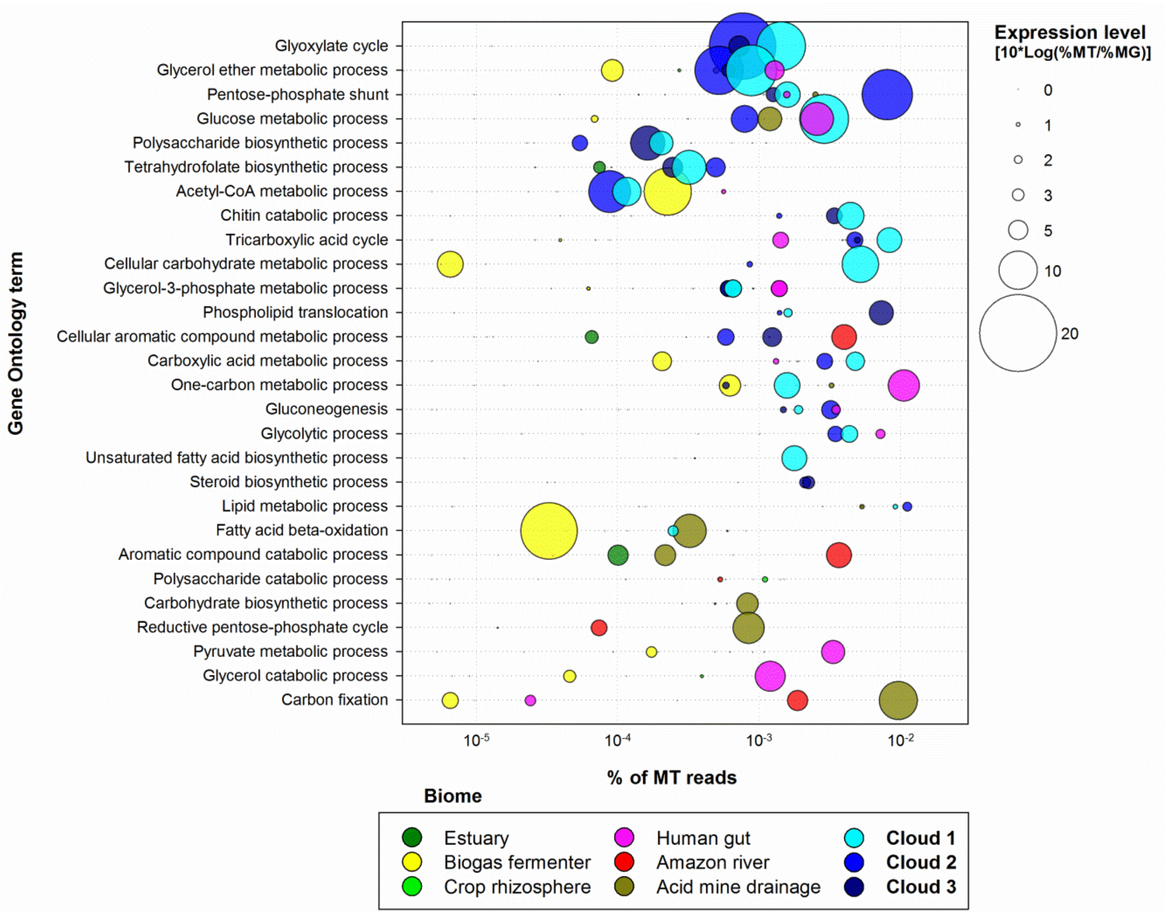

Figure S8: Biological processes related with **carbon metabolism** in clouds, compared with other environments, ordered by their summed expression level in clouds (cumulated bubble size). See legend of **Figure S4** for further details.

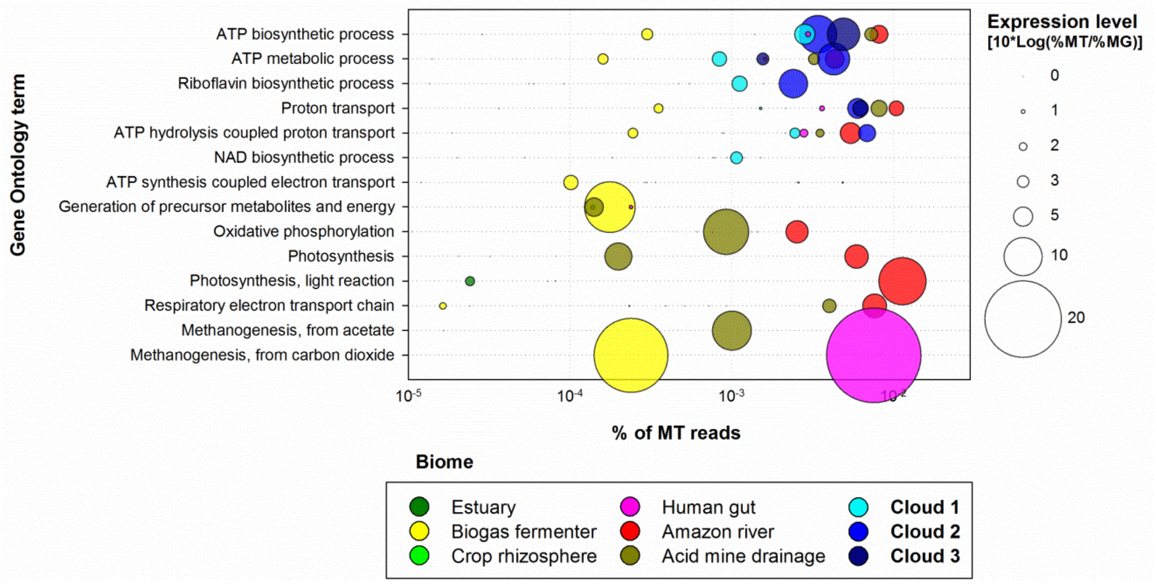

Figure S9: Biological processes related with **energy and redox metabolisms** in clouds, compared with other environments, ordered by their summed expression level in clouds (cumulated bubble size). See legend of **Figure S4** for further details.

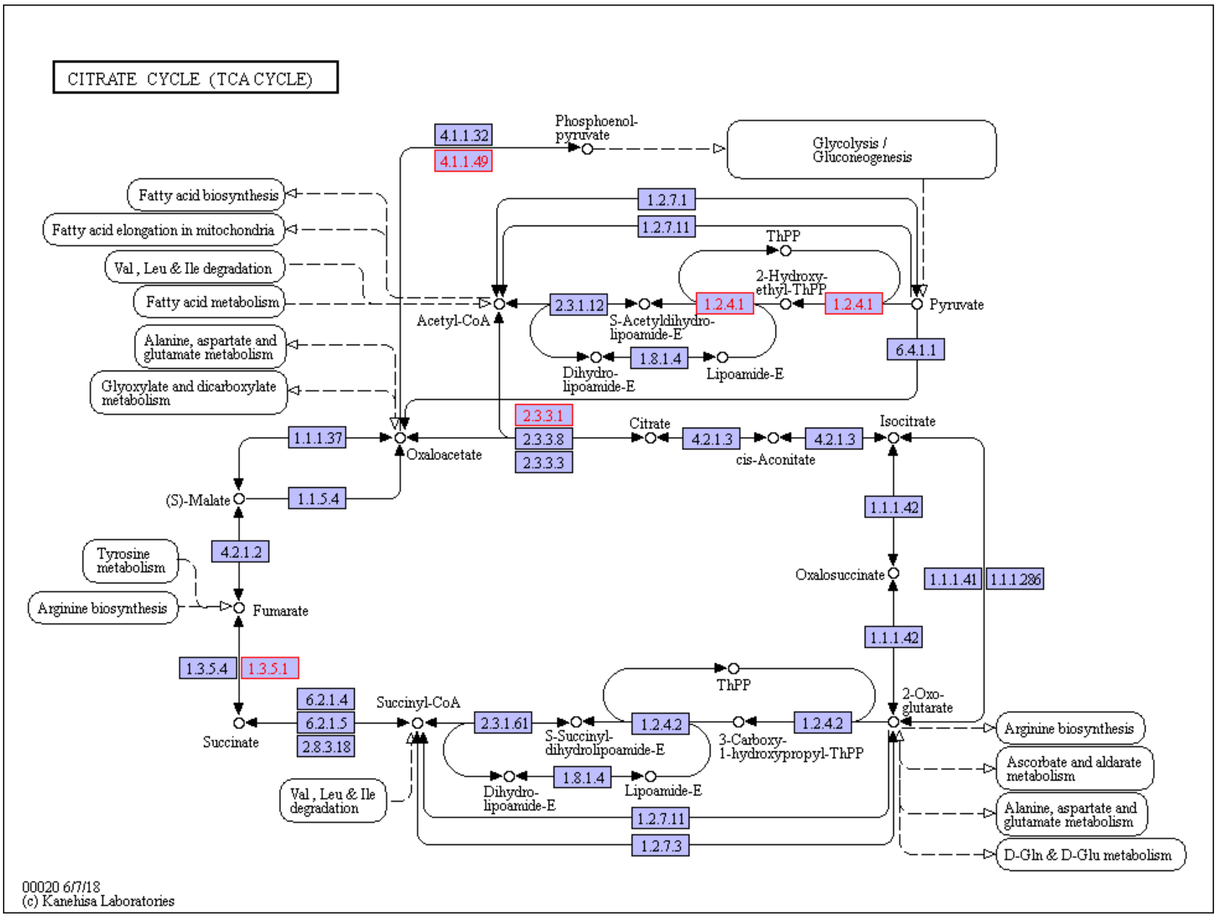

Figure S10: The TCA cycle metabolic pathway and known E.C. numbers of the enzymes involved, from KEGG [2–4]. The enzymes detected at high level in cloud MT are indicated in red. E.C.1.2.4.1: pyruvate dehydrogenase (GO:0004739); E.C.1.3.5.1: succinate dehydrogenase (GO:0008177 and GO:0000104); E.C.4.1.1.49: phosphoenolpyruvate carboxykinase (ATP) (GO:0004611-4612); E.C.2.3.3.1: citrate synthase (GO:0004108).

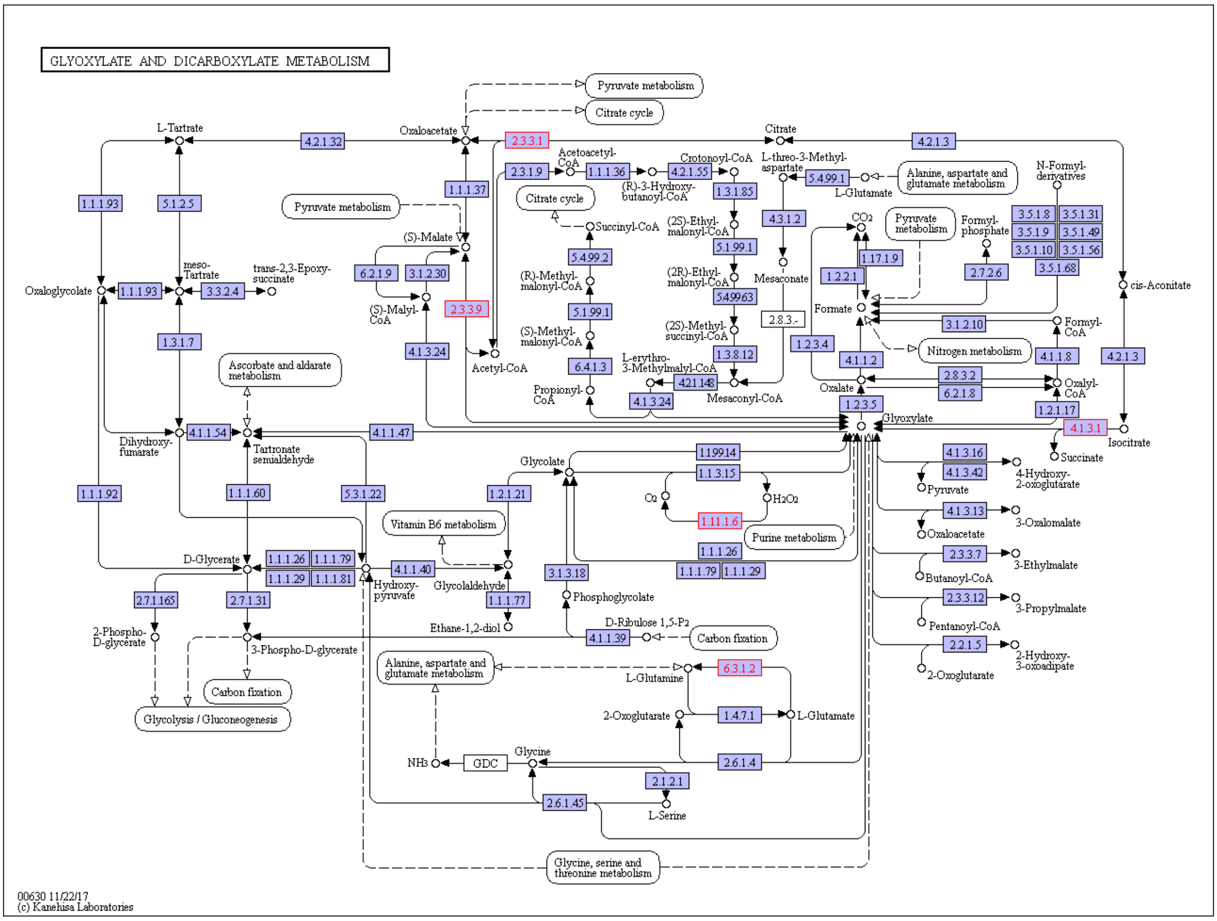

Figure S11: The glyoxylate cycle metabolic pathway and known E.C. numbers of the enzymes involved, from KEGG [2–4]. The enzymes detected at high level in cloud MT are indicated in red. E.C.1.11.1.6: catalase (GO:0004096); E.C.2.3.3.1: citrate synthase (GO:0004108); E.C.2.3.3.9: malate synthase (GO:0004474); E.C.4.1.3.1.: isocitrate lyase (GO:0004451); E.C.6.3.1.2.: glutamate-ammonia ligase (GO:0004356).

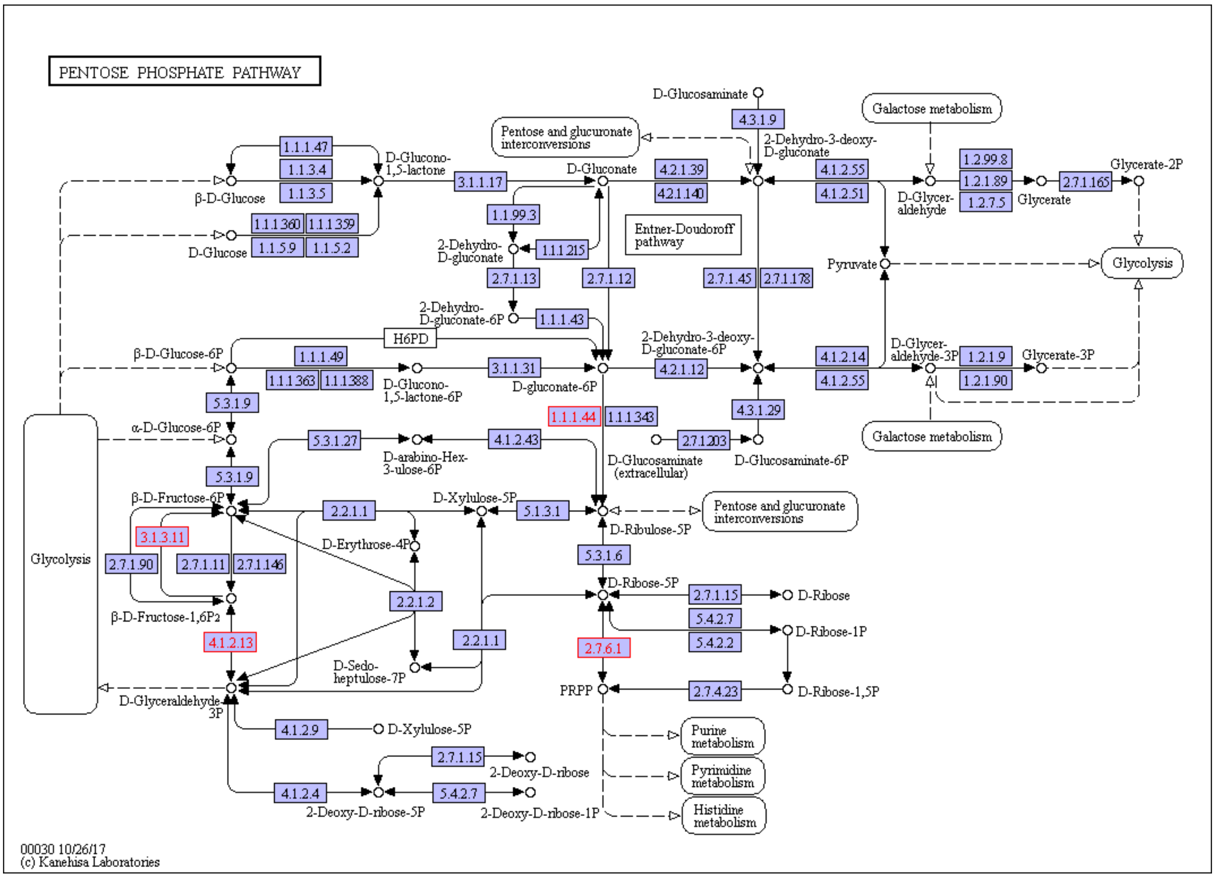

Figure S12: The pentose phosphate metabolic pathway and known E.C. numbers of the enzymes involved, from KEGG [2–4]. The enzymes detected at high level in cloud MT are indicated in red. E.C. 1.1.1.44.: phosphogluconate dehydrogenase (NADP+ dependent, decarboxylating (GO:0004616); E.C. 2.7.6.1.: ribose phosphate diphosphokinase (GO:0004749); E.C. 3.1.3.11: fructose 1,6-bisphosphate 1-phosphatase (GO:0042132); E.C. 4.1.2.13: fructose-bisphosphate aldolase (GO:0004332).

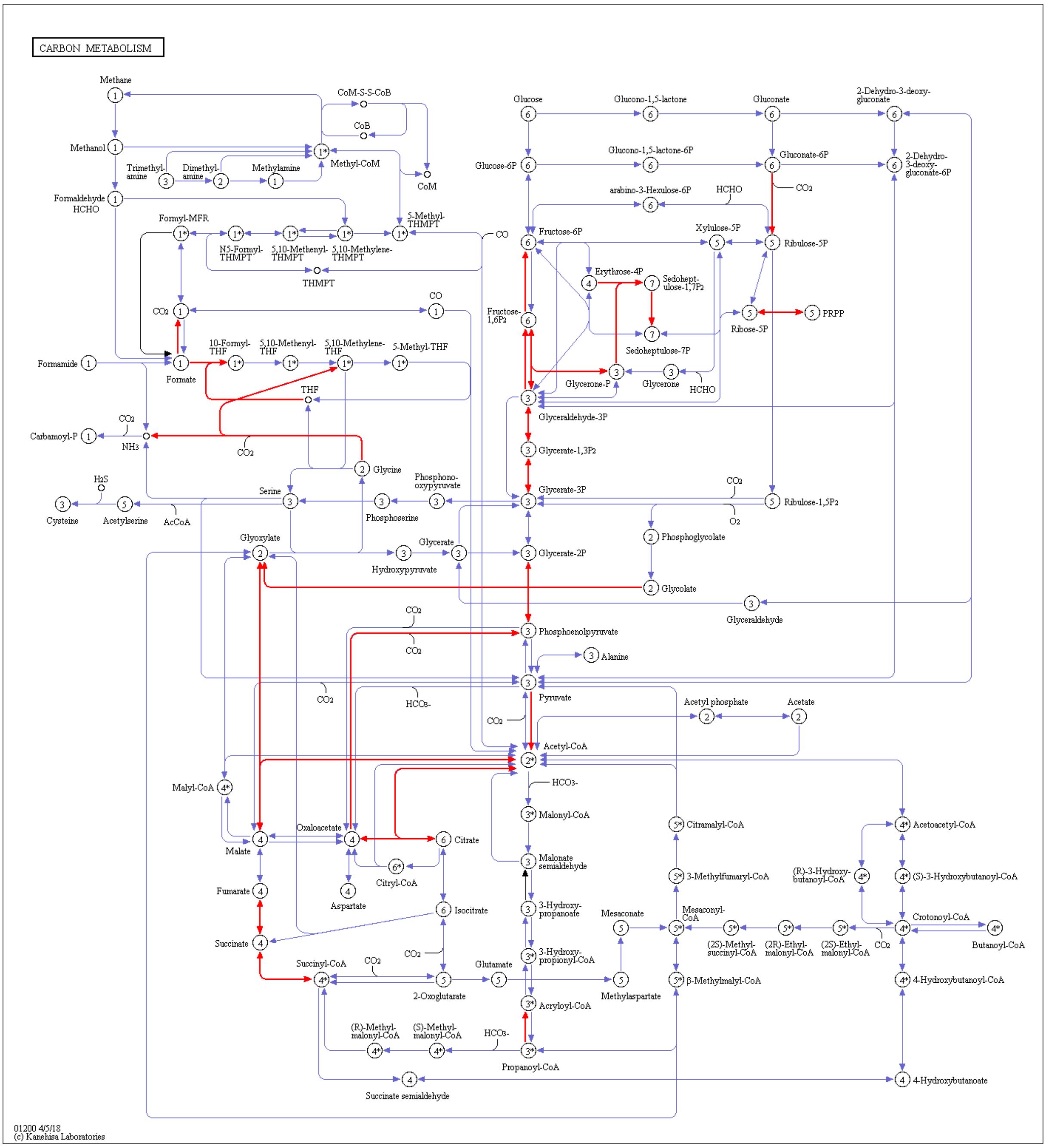

Figure S13: Summary of the carbon metabolic pathways detected overexpressed in the cloud water microbial communities, based on molecular function GO terms and the associated E.C. numbers in KEGG [2–4]. Several molecular functions remained unaffiliated with a specific enzyme so this is not exhaustive and other routes probably occurred.

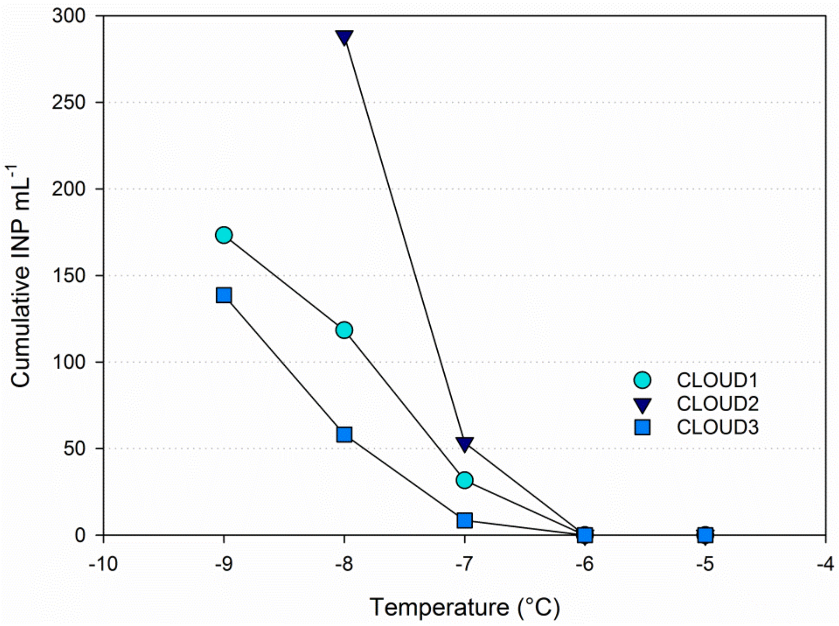

Figure S14: Cumulative ice nucleating particle concentration in the samples, determined by droplet freezing assay similarly as in Joly et al. (2014) [5], from 32 droplets of 20 µL.

Supplementary references:

1. Draxler, R. R. & Rolph, G. D. HYSPLIT (HYbrid Single-Particle Lagrangian Integrated Trajectory) Model access via NOAA ARL READY Website (http://ready.arl.noaa.gov/HYSPLIT.php). *NOAA Air Resources Laboratory, Silver Spring, MD* (2013).
2. Kanehisa, M., Sato, Y., Furumichi, M., Morishima, K. & Tanabe, M. New approach for understanding genome variations in KEGG. *Nucleic Acids Res.* **47**, D590–D595 (2019).
3. Kanehisa, M., Furumichi, M., Tanabe, M., Sato, Y. & Morishima, K. KEGG: new perspectives on genomes, pathways, diseases and drugs. *Nucleic Acids Res.* **45**, D353–D361 (2017).
4. Kanehisa, M. & Goto, S. KEGG: kyoto encyclopedia of genes and genomes. *Nucleic Acids Res.* **28**, 27–30 (2000).
5. Joly, M. *et al.* Quantification of ice nuclei active at near 0 °C temperatures in low-altitude clouds at the Puy de Dôme atmospheric station. *Atmospheric Chemistry and Physics* **14**, 8185–8195 (2014).
6. Fortunato, C. S. & Crump, B. C. Microbial Gene Abundance and Expression Patterns across a River to Ocean Salinity Gradient. *PLOS ONE* **10**, e0140578 (2015).

7. Bremges, A. *et al.* Deeply sequenced metagenome and metatranscriptome of a biogas-producing microbial community from an agricultural production-scale biogas plant. *GigaScience* **4**, 33 (2015).
8. Franzosa, E. A. *et al.* Relating the metatranscriptome and metagenome of the human gut. *Proc. Natl. Acad. Sci. U.S.A.* **111**, E2329-2338 (2014).
9. Guo, J. Rhizosphere metagenomics of three biofuel crops. (Michigan State University, 2016).
10. Satinsky, B. M. *et al.* The Amazon continuum dataset: quantitative metagenomic and metatranscriptomic inventories of the Amazon River plume, June 2010. *Microbiome* **2**, 17 (2014).
11. Chen, L. *et al.* Comparative metagenomic and metatranscriptomic analyses of microbial communities in acid mine drainage. *The ISME Journal* **9**, 1579–1592 (2015).
